# IMPACT OF OBESITY ON FUNCTIONING IN SOCIETY – OQI-3, A NEW TOOL PILOT STUDY RESULTS

**DOI:** 10.13075/ijomeh.1896.02547

**Published:** 2025

**Authors:** Monika Szkultecka-Dębek, Marta Bem, Aleksandra Gradowska, Mariola Drozd

**Affiliations:** 1 University of Social Sciences, Faculty of Applied Sciences, Warsaw, Poland; 2 Qualitas Vitae Institute, Foundation, Warsaw, Poland; 3 SWPS University, Warsaw, Poland; 4 Medical University of Lublin, Department of Humanities and Social Medicine, Lublin, Poland

**Keywords:** obesity, quality of life, social functioning, work environment, instrument, daily functioning

## Abstract

**Objectives:**

The objective was to develop a tool enabling better understanding of obesity impact on social functioning with focus on daily functioning and work related activities.

**Material and Methods:**

*Obesity Impact on Functioning in Society Questionnaire* (OQI-3) was developed for use among adult population with obesity disease. It focuses on daily functioning, work related activities and emotions related to work environment social relations. The 3-part questionnaire combining qualitative and quantitative methods is gender specific and it was validated among 41 adult patients with obesity in Poland. The first part is a vignette examining patients' projected attitudes and emotions. The second is an open question related to daily activities mostly affected by obesity. The third part uses a Likert scale to assess the degree of difficulty in performing daily activities consisting of 11 actions taken in everyday life. Respondents select 1 of the answers on the scale to indicate the obesity influence on the mentioned activities.

**Results:**

The qualitative parts provided information on attitudes and emotions, where mostly negative attitudes and emotions were mentioned. The most frequently mentioned were physical activity, walking up the stairs, housework and activities related to patients' image. The quantitative part identified 2 factors (1 – relations in the further social environment, 2 – functioning in a close environment). The Likert scale was recoded for each statement as follows: 1 – 3; 2 – 1; 3 – 2; 4 – 4; 5 – 5 and Cronbach's α value was calculated confirming scale reliability. It equals 0.874 for the total of items.

**Conclusions:**

The OQI-3 is innovative and combines various research techniques to verify people suffering from obesity well-being and disease impact on social functioning. The pilot study has proven its internal consistence. However the modified tool based on the pilot results should be tested and validated on a larger study group. Guidelines for results interpretation should be developed with the final instrument version.

## Highlights

- There is an unmet need for better understanding obesity impact on patients' social functioning.
- *Obesity Impact on Functioning in Society Questionnaire* (OQI-3) focuses on obesity impact on daily functioning and work related activities.
- Preliminary analyses suggest good reliability and internal consistence for 2 factors.
- *Obesity Impact on Functioning in Society Questionnaire* (OQI-3) is an innovative tool measuring obesity impact on patients' social functioning.

## INTRODUCTION

According to World Health Organization (WHO), within the last 50 years, the number of people with obesity worldwide tripled [1]. World Health Organization reports state that obesity has reached epidemic proportions globally, with at least 2.8 million people dying each year as a result of being overweight or obese and based on the Organisation for Economic Co-operation and Development (OECD) data, life expectancy in the EU has shortened by almost 3 years due to overweight and obesity [2,3]. Based on the WHO global data from 2022, 43% of adults aged ≥18 years were overweight and 16% were living with obesity [2]. The World Obesity Federation projects a rise in the number of adults suffering from obesity from 0.81 billion in 2020 to 1.53 billion in 2035 [4]. According to a report relating to data from 2016 and published by the National Health Fund (NHF) in Poland, among people aged ≥20 years, 53% of women and 68% of men were overweight, and 23% of women and 25% of men were obese [5]. The number of people with obesity disease in Poland reaches approx. 9 million. The estimation was done by the Supreme Audit Office and it is based on the data from the Central Statistical Office and the NHF. According to the NHF, in the years 2020–2022, almost 800 000 people were treated for obesity in primary care and emergency care and they received a total of 2.2 million services [6]. Defining obesity as body mass index (BMI) ≥30 kg/m^2^ WHO analysis revealed that in 2016 13% of adult population fulfilled that criterion meaning that they suffered from obesity, both men and women (11% and 15%, respectively) and according to the Polish Public Opinion Research Center in 2014, 34% were overweight and 17% suffered from obesity disease in Poland [7].

Obesity impact on health is considerable and by being one of the metabolic syndrome components it is often correlated with concomitant diseases such as type 2 diabetes mellitus or arterial hypertension. There are studies demonstrating obesity impact on overall quality of life (QoL) and mental condition. Quality of life is a complex term influenced by both internal and external factors, and the different dimensions of QoL might be affected among others by chronic diseases, such as obesity [1]. It is worth mentioning that published research confirm that people with obesity show higher sensitivity and more emotional reactions to stressful situations. According to the researchers, people with obesity react to different everyday life situations with more intensity than those with a normal body weight [8]. Among the researchers there are examples of examining the QoL and life satisfaction by analyzing the relationship between body image and self-assessment as well as depression symptoms incidence among patients with obesity using different psychometric scales, like the *Tennessee Self-Concept Scale* developed by Fitt, *Beck Depression Inventory, Quality of Life and Satisfaction with Life Scale*. The findings from another study have shown that overweight and obese persons demonstrated higher self-awareness within areas which were not related to physical appearance, especially those related to the personal and social aspects, which is probably to protect themselves from a negative perception and from low self-confidence [9]. It is worth mentioning that nowadays the disease becomes more often an issue within younger population and one of the studies conducted among children and adolescents explored the impact of excessive body weight on QoL using the *Pediatric Questionnaire of Quality of Life*. Authors concluded that obesity affects negatively the health-related QoL of the studied group irrespectively of their gender [10]. Researchers also pointed out the consequences of obesity in relation to children and adolescents, observing an increase of cardiovascular risk factors, premature mortality, as well as decreased fertility and worth to be mentioned negative socioeconomic effects [11].

A tool frequently used in research is the *Short Form 36* (SF-36) *Health Survey Questionnaire* which is a general questionnaire dedicated to measure QoL. Due to a wide scope the general QoL questionnaires enable obtaining results that are stable over time, however, due to the low sensitivity of the measurements, there is a probability of not detecting slight changes in the QoL. Often those QoL changes seen as generally insignificant may be of important clinical significance, especially from the patient's perspective. Multiple studies addressed general QoL, using tools for general population like those mentioned above SF-36 or the *World Health Organization Quality of Life Questionnaire* (WHO-QOL-BREF) in different settings [12,13]. A large patients' population with chronic diseases was studied to explore the impact of obesity on their QoL also with the use of SF-36 questionnaire. The findings confirmed the impact of obesity on QoL since those who were overweight or diagnosed with obesity had significantly lower adjusted physical function scores in comparison to the patients with the weight within normal range, the obese patients also had significantly lower adjusted general health perceptions scores and vitality scores [14].

It was demonstrated that SF-36 use in research focused on obesity requires a complementary tool since despite the fact that the results within the obese population confirm the correlation between BMI and poor health, the association is mostly within physical activity, and it is not related to dimensions like mental health or social functioning. Taking into consideration research findings it is recommended to use additionally a disease specific questionnaire, designed for patients suffering from obesity [15]. Among the specific questionnaires to measure QoL within the obese population there are several tools however some of them require further research with further validation and a better definition of the interpretability of existing instruments [16]. In relation to the specific instruments, it is worth mentioning the *Impact of Weight on Quality of Life* questionnaire (IWQOL). The tool was used in several studies, e.g., to measure the impact of obesity on QoL within middle aged women confirming the existence of positive significant correlation between body mass index and QoL within the studied group [17]. In order to assess QoL in case of morbid obesity an option for consideration is the *Laval Questionnaire*, developed by researchers from the Laval Hospital in Quebec City, Canada, however the tool was designed to be used specifically in clinical trials [18].

Based on the extensive research performed with the use of the variety of the existing of instruments for QoL measurement, there are still areas uncovered or situations with more details to be known in order to allow for better understanding of the specific disease, the impact it has on either patients QoL, the daily activities or on the social functioning of those suffering from the disease.

An option to gather additional insights from people suffering from obesity is to use specially designed vignettes. Using vignettes either in psychology or sociology field a hypothetical situation is described as potential scenario. Respondents by answering the questions are revealing perceptions, emotions, values or even social norms in relation to what the vignette describes. Such approach is also used to assess health related issues, sometimes within areas which are difficult for patients to be discussed openly or in the first person. In case of clinical use, the tool is applied to a single patient enabling elaboration regarding patients' scores in the QoL assessment [19]. It is worth mentioning that in psychology a tool that incorporates qualitative methods provide additional value and often is an additional module incorporated into the psychological questionnaires (e.g., *Strengths and Difficulties Questionnaire –* SDQ). The qualitative methods enable obtaining data from the respondents' deeper thoughts allowing for the internal experiences and their impact on everyday functioning analysis. Such methods, with open questions enable to capture and identify specific obstacles and needs that are not available through closed questions.

Moreover the qualitative data obtained in the study allow for the practical use of data by designing appropriate interventions used by practitioners. Qualitative methods are a perfect complement to quantitative data, which often explain the obtained results of statistical analyses. Qualitative methods allow for a more holistic approach to the problem. For future qualitative analysis of large samples, in further analyzes it is planned the use of a tool for coding data and changing their nature from qualitative scales to quantitative data. An option can be an analysis of qualitative data after dividing the sample into smaller groups.

In reference to the above, and since those who suffer from obesity often perceive stressful and potentially also daily situations differently than those people with their weight within normal range it might be worth to study the obesity disease impact on daily activities and patients' functioning in society and social perception of this group from the subjective point of view, where daily activities should be understood as performing everyday activities such as shopping, preparing meals or cutting nails.

In relation to the identified need for more insights in relation to people with obesity emotions, feelings and functioning in society, the aim of the research was to develop an instrument enabling the data collection, assessment and analysis. The developed tool's intention was to focus on a better understanding of obesity impact on social functioning in adult population in Poland with special attention to daily functioning, work related activities and working environment. The instrument might be used for research or as a tool supporting psychotherapist consultation for people with obesity, providing information on the current emotional state and main issues of concern in relation to the daily activities, work life and social life of the patients.

## MATERIAL AND METHODS

In order to verify the tools used for the assessment of QoL and daily functioning of obese patients a literature search was performed. The review consisted of a search of electronically available literature at PubMed and Google Scholar. The search was not limited for time and the used key words were “quality of life” and “obesity.”

Since the objective of the project was to develop a tool available in Polish to be used by health professionals during their interactions with their patients suffering from excessive weight, an important step was to identify the currently available instruments for specific use in case of obesity related QoL. The identification of the existing gaps and potential also need to be addressed.

A comprehensive analysis related to the already used tools and the available language versions of the QoL instruments enabled the process of designing of the new tool.

### Study design

The pilot study was aimed to reach adult patients, in a productive age, active economically, with diagnosed obesity (BMI ≥30 kg/m^2^) or overweighted (BMI 25–29.9 kg/m^2^). The planned number of respondents was min. 40. The study was performed at a private, specialized outpatient clinic in Warsaw, Poland, where patients with overweight and obesity were treated. The total pilot study period was between June 2023 – May 2024 and an Ethics Committee approval issued by the Ethics Committee at the University of Social Sciences, Warsaw, Poland, was obtained (No. 3/24). Before providing answers to the questionnaire patients were informed about volunteer participation, data anonymity, and the possibility of withdrawing from the study at any stage. The data were collected within a pilot group with the use of a new tool, 3-part questionnaire assessing the impact of obesity on social and daily functioning, *Obesity Impact on Functioning in Society Questionnaire* (OQI-3).

In addition to the developed instrument (OQI-3) a short questionnaire was used with a set of demographic questions in order to enable studied group description and to analyze patients' characteristics or to replicate the presented research.

### Research tool

The new questionnaire was created based on the experience of professionals working with patients with obesity and the identified need. The OQI-3 is a questionnaire, developed by Qualitas Vitae Institute Foundation, designed for self-administration as paper and pencil interview form, and it requires approx. 5–7 min to provide the answers. The tool consists of 3 main sections (2 qualitative and 1 quantitative), where the first is a vignette, followed by open questions related to respondent's observed daily activities impacted by excessive body weight, and the last section is a table with selected activities to be rated by the respondents. The main sections are preceded by the demographic data questions. The vignette is adjusted for gender, there are separate versions for females and males, both have 4 scenarios, all of them are related to work life, to the professional relations. The first scenario situates the respondent in a setting of a job search, the second and the third are focused on relations with peers (female vs. male colleagues), and the last scenario concerns the relationships with the direct superior or line manager at work. The questions are open and they are split into 2 parts. Firstly, the respondent is asked to describe in their own words the observed reactions of the people with whom they interact. Then, the second part of the question concerns respondents' feelings and what emotions appear in each of the described situations.

The second section of the OQI-3 is the open question related to daily activities. The respondent had to identify and provide information on the daily activities most affected and which of these activities cause more difficulties for the respondent on a daily basis due to obesity.

The third part of the tool consists of a list of activities provided in the form of a table, which are to be rated in terms of the impact of obesity on each activity. The rating is based on a Likert scale, allowing for the following answers: high impact, medium impact, low impact, no impact, neutral. The Likert scale was recoded as follows for each statement: 1 – 3, 2 – 1, 3 – 2, 4 – 4, 5 – 5. The indicators will be created in such a way that factors 1 and 2 will be created as a summary indicator of items that will correspond to high factor loadings for each of the factors. The higher the result, the higher intensity of the tested item.

**Figure 1.**
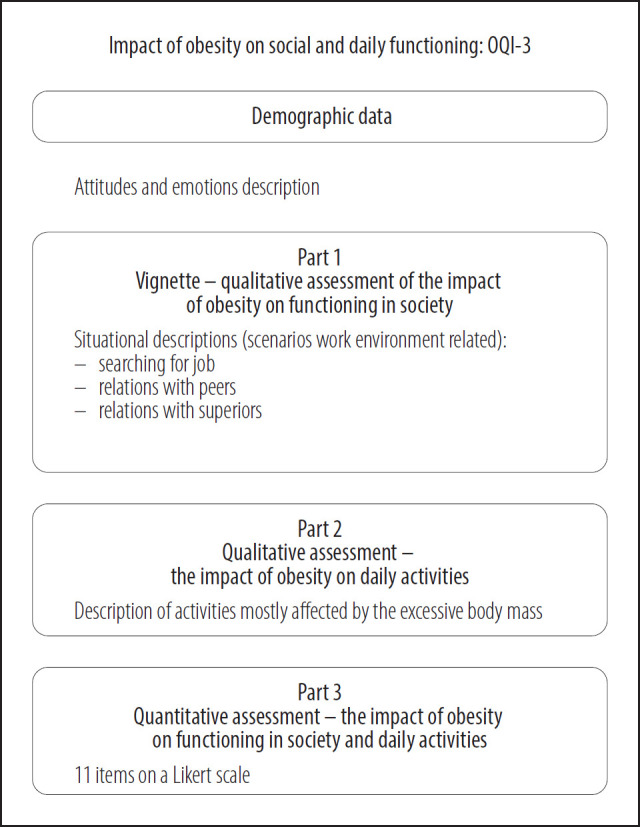
The *Obesity Impact on Functioning in Society Questionnaire* (OQI-3) outline

The questionnaire outline is presented in Figure 1.

### Statistical analysis

The statistical analysis was planned for a pilot of the min. 40 respondents and to be performed with the IBM SPSS program, v. 29. In order to ensure reliability of the quantitative part Cronbach's α value was calculated. The qualitative parts were planned to be analyzed with expert judges by grouping the answers into categories. The expert judges were male and female adults, professionally active, without excessive body weight.

## RESULTS

### Study group characteristics

A total of 41 respondents participated in the pilot study and the results for each of the OQI-3 sections were statistically analyzed using SPSS program.

The studied group characteristics is summarized in Table 1.

**Table 1.** Characteristics of the study group of 41 adult patients with obesity, specialized outpatient clinic in Warsaw, Poland, June 2023 – May 2024

Variable	Participants (N = 41)	
	n	%
Gender		
female	27	65.9
male	14	34.1
Age		
18–20 years	1	2.4
21–30 years	2	4.9
31–40 years	6	14.6
41–50 years	13	31.7
51–60 years	15	36.6
>60 years	4	9.8
Place of residence		
village	6	14.6
city	34	82.9
missing data	1	2.4
Education level		
primary	2	4.9
high school	13	31.7
university	26	63.4

Among all respondents there were 36 with BMI >30 kg/m^2^ and their average BMI was 38.95 kg/m^2^ (SD = 7.21). The characteristics related to respondents' body parameters as declared by patients are presented in Table 2.

**Table 2.** Body parameters analysis based on the 41 patients with obesity, specialized outpatient clinic in Warsaw, Poland, June 2023 – May 2024

Variable	M	Me	SD	Min.	Max
Heigh [cm]	171.78	169.00	11.95	155.00	197.00
Weight [kg]	111.20	108.00	27.67	75.00	189.00
Waist circumference [cm]^a^	113.88	110.00	20.18	90.00	160.00
BMI [kg/m^2^]	37.53	36.34	7.79	25.35	58.33

a N = 18.

### OQI-3

#### Part 1: Work environment related vignettes – qualitative results

The first part of the questionnaire examines the projected attitudes and emotions of people with weight problems and consisted of a vignette with 4 scenarios within work environment. The first scenario described a situation where a mid-aged person was searching for a job and the respondents were asked to describe what behaviors they observe during the interaction with the recruiter, followed by naming the emotions the candidate for the job may feel. Similar task was given to the respondents for the next case, where the scenario described the relations within work environment with female and male peers as well as with the line manager or their superiors.

The first section of the tool had a descriptive nature, therefore 4 independent expert judges were asked to group the answers into categories. In 3 cases the obtained categories were similar. Two judges described the observed attitude groups as negative, positive and neutral. One of the judges proposed categories as nastiness, positive attitude and negative attitude, while the fourth one proposed to group the attitudes as private relations, professional relations, image or appearance related.

In relation to the emotions the expert judges identified categories as follows: negative, positive and neutral emotions. In 1 case the judge suggested to include into negative emotions a category of complexes, while another instead of neutral emotions identified a group of emotions related to sadness.

Within the attitudes observed the most frequent were the negative attitudes, e.g., at first scenario respondents pointed out to scanning their body with eyes, contemptuous glances, less interest in the person and what they have to say, strange glances, critical judgement of the external appearance – not necessarily verbalized. In terms of relationships at work also the negative attitudes were quite frequent, like mocking, “they call me fat,” comments about healthy life and physical activity, providing “good tips,” 2 however some of the respondents declared neutral attitude or even supportive and motivating behavior. The last scenario was focused on the relation with the superiors at work and mostly there were neutral attitudes observed, no special reactions, maybe not assigning the person to heavy physical work.

Among emotions the most frequently named in all scenarios were frustration, sadness, regret, sense of discrimination, shame.

#### Part 2: Impact of obesity on daily activities – qualitative results

The second part of the tool was an open question related to daily activities enabling to collect patients' answers and with the support of expert judges to create a statements cafeteria which will be included in the final instrument. The respondent was asked to identify those activities which are influenced and more difficult to perform due to the body mass.

**Figure 2.**
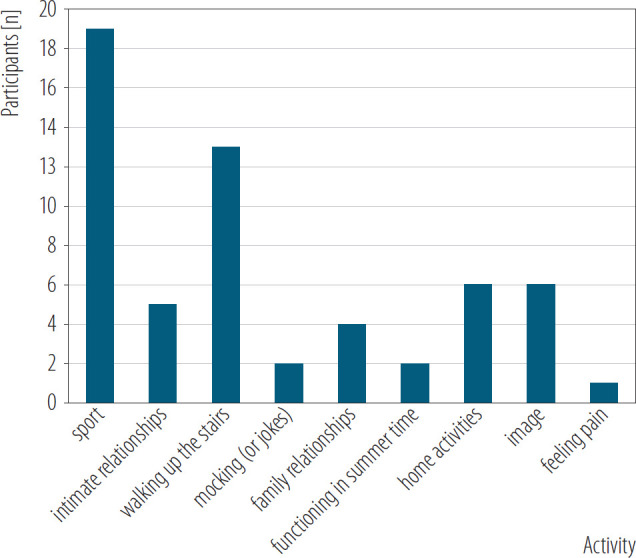
Activities impacted by excessive body weight of the 41 patients with obesity, specialized outpatient clinic in Warsaw, Poland, June 2023 – May 2024

In order to analyze this information, the group of expert judges were asked to group the answers by categories and the proposed categories were as follows. The first judge split the answers into: relations, physical condition, sexual activities, duties; the second one into physical condition, sexual activities/relations, everyday life; the third one proposed physical condition, health, image, everyday life; and the fourth one physical condition, relations, everyday life and nice things. Summarizing the daily activities were grouped as physical condition, relations, everyday life, however more detailed answers are represented in Figure 2.

The most frequently mentioned by respondents as affected area of their daily functioning due to obesity it was physical activity, walking up the stairs, housework and activities related to their image. The less frequently mentioned were feeling pain, functioning during summer and jokes (related to their condition).

#### Part 3: Impact of obesity on daily activities – quantitative results

The third part of the tool consisted of a table with a list of activities and Likert scale for assessment of each activity impact on daily life was used. The Likert scale was recoded as follows for each statement: 1 – 3; 2 – 1; 3 – 2; 4 – 4; 5 – 5 and the results of the statistics to test the reliability of the questions are presented at the tables below. For a ratio of 41:11, the result (3.73:1) does not meet the minimum recommendation of 5:1 by Nunnally [20] and Kline [21]. However, this is a pilot of the tool, not a final psychometric analysis. The presented results constitute the first of several stages of analysis of the tool parameters. Due to the limited number of observations, an exploratory factor analysis (EFA) was performed.

Additionally, the factor analysis was performed for accuracy using the main components method.

The existence of 2 orthogonal factors was demonstrated. Varimax rotation was used as a correction for factors' orthogonality. The analysis results with the matrix of the rotated components is presented in Table 3, where factor 1 is related to functioning in the social environment (relations in the further social environment) and factor 2 relates to functioning in a close environment. The Kaiser-Mayer-Olkin (KMO) test result of 0.732 indicates good sample quality, suggesting that the data are suitable for factor analysis. Moreover, the result of Bartlett's test of sphericity (χ^2^ = 194.532, p < 0.001) confirms the existence of significant correlations between the variables, which also indicates the adequacy of the data for factor analysis. In principal component analysis (PCA), the first 2 components explain a total of 63.63% of the variance in the data (component 1 – 46.16%, component 2 – 17.47%). After rotation, the cumulative percentage of explained variance for these 3 components is 57.73%, with the first component explaining 31.14% of the variance and the second 26.59%.

**Table 3.** Rotated component matrix of the *Obesity Impact on Functioning in Society Questionnaire* (OQI-3), specialized outpatient clinic in Warsaw, Poland, June 2023 – May 2024

OQI-3 item	Component	
	factor 1	factor 2
Shopping		0.717
Housework		0.644
Contacts with other	0.781	
Free time	0.741	
Social life	0.709	
Relations with partner		0.773
Relations with family		0.750
Relations with friends		0.720
Practicing sports	0.820	
Physical activity	0.801	
Perception in the work environment	0.674	

Factor 1 – relations in the further social environment; factor 2 – functioning in a close environment.

### Reliability analysis

In order to assess the reliability for the entire scale the Cronbach's α value was calculated for the total of items (11 items) in section 3 of the instrument and it equals 0.874. The questions of the third section of the tested tool were analyzed in detail and it was considered removing questions to increase the reliability of the instrument. However, the analysis shown that removing any of the questions will have no significant impact on the scale reliability (Table 4).

**Table 4.** Total item statistics results of the *Obesity Impact on Functioning in Society Questionnaire* (OQI-3), specialized outpatient clinic in Warsaw, Poland, June 2023 – May 2024

OQI-3 item	Scale mean after item removal	Scale variance after item removal	Item correlation (total)	Cronbach's α after item removal
Shopping	30.433	85.490	0.249	0.885
Housework	30.000	80.759	0.447	0.872
Contacts with others	30.033	74.861	0.638	0.859
Free time	29.868	77.292	0.649	0.859
Social life	30.133	73.085	0.788	0.848
Relationship with partner	30.333	72.023	0.713	0.853
Relationship with family	31.000	77.310	0.605	0.862
Relationship with friends	31.100	78.714	0.607	0.862
Practicing sports	28.868	81.016	0.568	0.865
Physical activity	28.868	82.671	0.403	0.874
Perception in the work environment	26.184	0.651	0.552	0.861

The mean (M) values for each factor, as well as the overall average, were calculated. For factor 1 M ± standard deviation (SD) 19.097±6.123, while for factor 2 12.775±5.161. The overall value was 31.561±9.808.

The factor 1 indicator (relations in the further social environment) is a summary indicator of the following items: contacts with others, free time, social life, practicing sports, physical activity, perception in the work environment. The factor 2 indicator (functioning in a close environment) is a summary indicator of the following items: housework, shopping, relations with the partner, relations with family and relations with friends.

Both factors, 1 and 2 have a normal distribution (p > 0.05, Shapiro-Wilk test), and the level of correlation between the factors was checked. The analysis showed that the relationship is significant and low (r = 0.421, p = 0.007, R = 17.7%). The results indicate that there is a significant relationship between the 2 subscales (factor 1 and factor 2), the relationship is low (moderate correlation 0.421) which partially explains a low variability (17.7%).

It is worth keeping the assumed division of the scale into the 2 factors, due to the PCA value. The first 2 components explain a total of 63.63% and the low correlation between the examined factors. Determining 2 factors allows to capture subtle differences between different aspects of the examined feature, i.e., different psychological dimensions. The reliability analysis for factor 1 (6 items) obtained Cronbach's α 0.876, therefore confirming the questionnaire reliability.

Reliability analysis for factor 2 (5 items) resulted in Cronbach's α at 0.806 and by that the tool reliability is confirmed.

Additionally, tests were performed to validate the reliability and impact on Cronbach's α after an item removal. The statistics shows that by removing a question there is no observed further increase of the reliability.

## DISCUSSION

The OQI-3 instrument aimed to enable a better understanding of obesity impact on social functioning with a special focus on patient' daily functioning and work related activities. The reason for centering the attention of the research on obesity was that it is becoming a serious health issue from the worldwide perspective and currently it is considered a lifestyle disease.

Obesity as a disease affects not only the body, but often it is also a psychological problem and as such it requires appropriate treatment [22]. Several literature reviews analyzing patients' data worldwide found that within the group of people with obesity and extreme obesity 20–60% of them suffer from a psychiatric illness [23].

According to the clinical recommendations for management of patients with obesity published in 2022, psychological treatment is one of the important elements of patients' therapy [24]. The performed pilot study with OQI-3 revealed mostly negative attitudes and emotions among respondents with diagnosed obesity.

Across a number of studies, it was demonstrated that obesity correlates with people QoL. Patients suffering from the disease have worse QoL, mostly due to physical and occupational functioning [24–28]. In the conducted pilot study, patients also declared worse physical functioning and functioning in the work environment.

The newly developed tool (OQI-3) is an innovative instrument since it applies to professionally active people who suffer from overweight or obesity. It takes into consideration not only the daily activities but also the social aspects and the work environment of people being in productive age. Previous research conducted on people suffering from obesity concerned people outside the work environment. Within the conducted pilot research focused on the tool validation it can be observed already that the instrument will provide excellent assistance to psychotherapists in planning their work with patients suffering from obesity not only in order to improve patients' well-being and make the treatment more effective, but also to make their return to work easier and support in living a fulfilling life. Additionally, it might be a helpful tool in developing support programs and social campaign against existing stereotypes regarding people with obesity.

It is worth mentioning that by working with the new instrument, researchers, psychotherapists and other specialists, during treatment sessions with people suffering from obesity, they can “hear” the voice of the respondents regarding their subjective feelings and emotions related to social contacts. However, it is worth mentioning that for each group such as children, teenagers, young adults, people of working age, people of post-working age the tools examining the QoL must be slightly different. In each group, different factors contribute to the level of QoL and each of them has different possibilities to work with the disease. Specialists working with ‘people with obesity should be able to diagnose the patient's needs and use the appropriate instrument.

### Study limitations

The study was a pilot conducted on a small sample and requires a next phase in a larger population in order to confirm preliminary findings. Due to the small sample, the authors are conscious of a lower stability of the results and as a result, factor solutions may be less reliable. However, in the first stage of the pilot study, they can be considered as sufficient.

The qualitative parts of the questionnaire were open for respondents’ answering and in case of future research that section could be modified providing a listing of possible items based on those obtained within the pilot.

The data pertaining to height and weight were declarative, which could have influenced the classification of patients according to BMI as overweight rather than obese. In the future the next study phase could compare results for overweight and obese population. Since there is no similar instrument available it was also not possible the assessment of relevance by correlation with another tool.

## CONCLUSIONS

The newly developed tool is innovative, and it combines various research approaches and techniques to verify the well-being of people suffering from obesity and within the pilot study it has been proven to be internally consistent. The expert judges agreed on the answers' categories within the first 2 sections of the validated instrument. The third section was tested for reliability obtaining high value for Cronbach's α. During the pilot study some degree of difficulty in naming emotions by the respondents was observed. The vignette part requires minor modification, either by clarifying questions, adding examples or by providing multiple choice answers options to first and second section. The modified tool should be tested and validated on a larger study group and guidelines for results interpretation should be developed with the final instrument version.

## References

[1] World Health Organization [Internet]. Geneva: WHO; 2024 [cited 2024 Aug 4]. Obesity and overweight. Available from: http://www.who.int/news-room/fact-sheets/detail/obesity-and-overweight

[2] World Health Organization [Internet]. Geneva: WHO; 2021 [cited 2024 Aug 4]. Obesity. Available from: https://www.who.int/news-room/facts-in-pictures/detail/6-facts-on-obesity.

[3] Business Insider. [Internet]. Ringier Axel Springer Polska; 2023 [cited 2024 Aug 4]. Most Poles are overweight. This is unhealthy as well as costly. Available from: https://businessinsider.com.pl/wiadomosci/wiekszosc-polakow-ma-nadwage-to-niezdrowe-oraz-kosztowne/mxtt611. Polish.

[4] The World Obesity Federation [Internet]. WOF; 2024 [cited 2025 Jan 21]. World Obesity Atlas 2024. Available from: https://www.worldobesity.org/news/world-obesity-atlas-2024

[5] Narodowy Fundusz Zdrowia. [Internet]. Szczecin: NFZ; 2019 [cited 2024 Aug 4]. Prezentacja raportu „Cukier, otyłość – konsekwencje”. Available from: https://www.nfz-szczecin.pl/dla_news_2168_prezentacja_raportu_bdquocukier_otylosc_ndash_konsekwencjerdquo.htm. Polish.

[6] Najwyższa Izba Kontroli. [Internet]. Warsaw: NIK; 2024 [cited 2024 Aug 4]. [Obesity prevention and treatment have outgrown the system (press conference transcript)]. Available from: https://www.nik.gov.pl/aktualnosci/coraz-wiecej-osob-z-choroba-otylosciowa-coraz-dluzsze-kolejki.html. Polish.

[7] Jung A. [Obesity – a lifestyle disease]. Pediatr Med Rodz 2014;10(3):226–32. 10.15557/PiMR.2014.0025. Polish.

[8] Kłósek P. [The relationships between psychological stress and obesity]. Forum Med Rodz. 2016;10(3). Polish.

[9] Bertrandt K. [Self-assessment vs. quality of life and occurrence of depression symptoms among overweight and obese persons in comparison to persons of normal weight]. Probl Hig Epidemiol. 2011;92(4):783–7. Polish.

[10] Sola M, Gajewska E, Manikowski W. [The influence of obesity on health-related quality of life among girls and boys]. Now Lek. 2012;81(4):321–9. 10.21784/IwP.2020.011. Polish.

[11] Gawlik A, Zachurzok-Buczyńska A, Małecka-Tendera E. [Complications of obesity in children and adolescents]. Endokrynol Otył Zab Przem Mat. 2009;5(1):19–27. Polish.

[12] Gnacińska-Szymańska M, Dardzińska A, Majkowicz M, Małgorzewicz S. [The assessment of quality of life in patients with excessive body mass using WHOQOL-BREF form]. Endokrynol Otył Zab Przem Mat. 2021;8(4):136–42. Polish.

[13] Zielińska-Więczkowska H, Budnik M. Analysis of the quality of life of overweight and obese patients with respect to body mass index and socio demographic factors. Farm Współ. 2016;9:110–6.

[14] Katz DA, McHorney CA, Atkinson RL. Impact of Obesity on HRQOL in Patients with Chronic Illness. J Gen Intern Med. 2000;15:789–96. 10.1046/j.1525-1497.2000.90906.x. 11119171 PMC1495614

[15] Beechy L, Galpern J, Petrone A, Das SK. Assessment tools in obesity – psychological measures, diet, activity, and body composition. Physiol Behav. 2012;107:154–71. 10.1016/j.physbeh.2012.04.013. 22548766 PMC7174029

[16] Duval K, Marceau P, Perusse L, Lacasse Y. An overview of obesity-specific quality of life questionnaires. Obesity Reviews. 2006;7:347–60. 10.1111/j.1467-789X.2006.00244.x. 17038129

[17] Kaur M, Singh S. Effect of obesity on quality of life in middle aged females. Int J Physiother. 2016;3(4):435–8. 10.15621/ijphy/2016/v3i4/111049.

[18] Therrien F, Marceau P, Turgeon N, Biron S, Richard D, Lacasse Y. The laval questionnaire: A new instrument to measure quality of life in morbid obesity. Health Qual Life Outcomes. 2011;9:66. 10.1186/1477-7525-9-66. 21843326 PMC3168398

[19] Szkultecka-Dębek M, Drozd M, Bem M, Kiepurska N, Mazur J. Is the vignette method used to assess quality of life in practice? Curr Issues Pharm Med Sci. 2015;28(1):8–12. 10.1515/cipms-2015-0032.

[20] Nunnally JC. An Overview of Psychological Measurement. In: Wolman BB, editor. Clinical Diagnosis of Mental Disorders. Boston, MA: Springer, 1978. pp. 97–146. 10.1007/978-1-4684-2490-4_4.

[21] Kline RB. Principles and practice of structural equation modeling. 3rd ed. New York: Guilford Press; 2011.

[22] Medycyna Praktyczna [Internet]. Mp.pl; 2024 [cited 2024 Aug 4]. Bąk-Sosnowska M. [The role of the psychologist in the treatment of obesity]. Available from: https://www.mp.pl/pacjent/dieta/odchudzanie/radypsychologa/63205,rola-psychologa-w-leczeniu-otylosci. Polish.

[23] Sarwer DB, Polonsky HM. The Psychosocial Burden of Obesity. Endocrinol Metab Clin North Am. 2016;45(3):677–88. 10.1016/j.ecl.2016.04.016. 27519139 PMC6052856

[24] Bąk-Sosnowska M, Białkowska M, Bogdański P, Chomiuk T, Gałązka-Sobotka M, Holecki M, et al. Zalecenia kliniczne dotyczące postępowania u chorych na otyłość 2022 – stanowisko Polskiego Towarzystwa Leczenia Otyłości. Med Prakt. 2022 May; Special Issue: 1–87. Polish.

[25] Fabricatore AN, Wadden TA, Sarwer DB, Faith MS. Health-related quality of life and symptoms of depression in extremely obese persons seeking bariatric surgery. Obes Surg. 2005; 15(3):304–9. 10.1381/0960892053576578. 15826461

[26] Fontaine KR, Barofsky I. Obesity and health-related quality of life [review]. Obes Rev. 2001;2(3):173–82. 10.1046/j.1467-789x.2001.00032.x. 12120102

[27] Kolotkin RL, Head S, Hamilton M, Tse C-KJ. Assessing impact of weight on quality of life. Obes Res. 1995;3(1):49–56. 10.1002/j.1550-8528.1995.tb00120.x. 7712359

[28] Kolotkin RL, Meter K, Williams GR. Quality of life and obesity. Obes Rev. 2001;2(4):219–29. 10.1046/j.1467-789x.2001.00040.x. 12119993

